# A Novel Alphabaculovirus from the Soybean Looper, *Chrysodeixis includens*, that Produces Tetrahedral Occlusion Bodies and Encodes Two Copies of *he65*

**DOI:** 10.3390/v11070579

**Published:** 2019-06-26

**Authors:** Robert L. Harrison, Daniel L. Rowley, Holly J. R. Popham

**Affiliations:** 1 Invasive Insect Biocontrol and Behavior Laboratory, Beltsville Agricultural Research Center, USDA Agricultural Research Service, Beltsville, MD 20705, USA; 2 AgBiTech, Fort Worth, TX 76155, USA

**Keywords:** soybean looper, *Chrysodeixis includens*, baculovirus, occlusion body, polyhedrin, lef-12, he65, DNA ligase 3

## Abstract

Isolates of the alphabaculovirus species, *Chrysodeixis includens nucleopolyhedrovirus*, have been identified that produce polyhedral occlusion bodies and infect larvae of the soybean looper, *Chrysodeixis includens*. In this study, we report the discovery and characterization of a novel *C. includens*-infecting alphabaculovirus, Chrysodeixis includens nucleopolyhedrovirus #1 (ChinNPV#1), that produces tetrahedral occlusion bodies. In bioassays against *C. includens* larvae, ChinNPV #1 exhibited a degree of pathogenicity that was similar to that of other ChinNPV isolates, but killed larvae more slowly. The host range of ChinNPV#1 was found to be very narrow, with no indication of infection occurring in larvae of *Trichoplusia ni* and six other noctuid species. The ChinNPV#1 genome sequence was determined to be 130,540 bp, with 126 open reading frames (ORFs) annotated but containing no homologous repeat (*hr*) regions. Phylogenetic analysis placed ChinNPV#1 in a clade with other Group II alphabaculoviruses from hosts of lepidopteran subfamily Plusiinae, including Chrysodeixis chalcites nucleopolyhedrovirus and Trichoplusia ni single nucleopolyhedrovirus. A unique feature of the ChinNPV#1 genome was the presence of two full-length copies of the *he65* ORF. The results indicate that ChinNPV#1 is related to, but distinct from, other ChinNPV isolates.

## 1. Introduction

The soybean looper, *Chrysodeixis includens* (Walker) (Lepidopera: Noctuidae), is a defoliating insect pest found throughout the Americas, from Quebec to southern South America [1]. The larvae of this moth are polyphagous and attack many crops but constitute a serious pest of soybean in the United States [2]. The soybean looper has recently surpassed the velvetbean looper (*Anticarsia gemmatalis*) as a primary lepidopteran pest of soybean in Brazil, a major soybean producer [3]. Efforts to control soybean looper populations have relied primarily on chemical insecticides, but the occurrence of resistance to many different insecticides has prompted the search for alternative methods of control [4,5,6].

Lepidopteran pests such as *C. includens* have been controlled with biopesticides based on viruses of the family *Baculoviridae* [7,8]. These insect-specific viruses possess large, circular double-stranded DNA genomes that are contained within virions composed of enveloped, rod-shaped capsids [9]. The virions in turn are assembled into distinctive occlusion bodies (OBs) that occur in a variety of shapes and sizes and can be harvested and applied as a biopesticide. The OBs consist primarily of a paracrystalline matrix formed from a single baculovirus-encoded protein that is synthesized in large quantities in infected host cells. This matrix dissolves in the alkaline environment of the larval host midgut, releasing the occluded virions so that they can initiate a lethal infection of the host. Most species of *Baculoviridae* occur in two genera, *Alphabaculovirus* and *Betabaculovirus*, consisting of viruses that exclusively infect larvae of Lepidoptera [10]. Much of what is known about baculoviruses comes from research with isolates of the type species *Autographa californica multiple nucleopolyhedrovirus* and also *Bombyx mori nucleopolyhedrovirus*. However, with the widespread availability of high-throughput sequencing instrumentation, many genomes of novel alpha- and betabaculoviruses isolated from a wide variety of lepidopteran host species worldwide have been determined and published in recent years.

Isolates of a baculovirus, Chrysodeixis includens nucleopolyhedrovirus (abbreviated ChinNPV) have been recovered from *C. includens* populations from Guatemala and Brazil [11,12]. Studies have demonstrated that these isolates have the potential for controlling *C. includens* infestations in the field [13,14], and a biopesticide based on ChinNPV has been registered for control of *C. includens* in Brazil [15] that is effective on populations resistant to two chemical insecticides with different modes of action [16]. Analysis of the genetic sequences for ChinNPV isolates [17,18,19] indicated that they were members of a single alphabaculovirus species, *Chrysodeixis includens nucleopolyhedrovirus*, which was created and ratified by the ICTV in 2016 [20].

This paper reports a new alphabaculovirus isolate from *C. includens*, ChinNPV#1. Although it has activity against *C. includens* larvae that is comparable to that of other ChinNPV isolates, the ChinNPV#1 OB morphology and genome sequence indicate that it is distinct from other ChinNPV isolates reported previously.

## 2. Materials and Methods

### 2.1. Virus Production and Isolation

The alphabaculovirus isolate ChinNPV#1 was originally extracted from larval cadavers of a South American *C. includens* population. Cadavers were ground in H_2_O, insoluble parts of the cuticle were removed by filtration through cheesecloth, and OBs were pelleted by centrifugation. Pellets were washed with 0.5% SDS then 0.5 M NaCl before final resuspension in H_2_O and storage at −20 °C until use.

ChinNPV-460 was sourced from the USDA Agricultural Research Service insect virus collection (Beltsville, MD, USA). Partial sequencing of the *lef-8*, *lef-9*, and *polh* loci of ChinNPV-460 indicated that it is a member of species *Chrysodeixis includens nucleopolyhedrovirus*, of which Pseudoplusia includens single nucleopolyhedrovirus-IE (PsinSNPV-IE) is the exemplar isolate [18].

To produce ChinNPV OB stocks, fifth instar *C. includens* larvae (Benzon Research, Carlisle, PA, USA), grown in 1-oz. plastic cups on artificial diet (Southland Products, Lake Village, AR, USA), were infected by pipetting 100 µL of a 1 × 10^6^ OBs/mL onto the diet surface. Larvae were maintained at 28 °C on a 14:10 light:dark cycle. Cadavers exhibiting symptoms of nuclear polyhedrosis were collected and OBs were extracted following the procedure described above. AcMNPV OBs were produced in a similar manner except AcMNPV was passaged in 4th instar *Trichoplusia ni* larvae (Benzon Research).

### 2.2. Electron Microscopy

All specimen preparation was performed at the Electron Microscopy Core Facility, University of Missouri, MO, USA. Unless otherwise stated, all reagents were purchased from Electron Microscopy Sciences (Hatfield, PA, USA). OBs were pelleted and fixed in 2% paraformaldehyde, 2% glutaraldehyde in 100 mM sodium cacodylate buffer pH = 7.35.

#### 2.2.1. Scanning Electron Microscopy (SEM)

Fixed samples were incubated overnight at 4 °C on cell culture treated coverslips to ensure adhesion. Next, fixed samples were rinsed with 100 mM sodium cacodylate buffer, pH 7.35 containing 130 mM sucrose. Secondary fixation was performed using 1% osmium tetroxide (Ted Pella, Inc. Redding, CA, USA) in cacodylate buffer using a Pelco Biowave (Ted Pella) operated at 100 Watts for 1 min. Specimens were next incubated at 4 °C for 1 h, then rinsed with cacodylate buffer and further with distilled water. Using the Pelco Biowave, a graded dehydration series (per exchange, 100 Watts for 40 s) was performed using ethanol. Samples were dried using the Tousimis Autosamdri 815 (Tousimis, Rockville, MD, USA) and samples were sputter coated with 5 nm of platinum using the EMS 150T-ES Sputter Coater. Images were acquired with a Hitachi S4700 scanning electron microscope (Hitachi High Technologies America, Dallas, TX, USA).

#### 2.2.2. Transmission Electron Microscopy (TEM)

Each sample was centrifuged at 2500 *g* and the resulting pellet was resuspended in HistoGel (Thermo Scientific, Kalamazoo, MI, USA). Next, fixed pellets were rinsed with 100 mM sodium cacodylate buffer, pH 7.35 (Sigma Aldrich, St. Louis, MO, USA) and 130 mM sucrose. Secondary fixation was performed using 1% osmium tetroxide (Ted Pella) in 2-ME buffer using a Pelco Biowave (Ted Pella) operated at 100 Watts for 1 min. Specimens were next incubated at 4 °C for 1 h, then rinsed with cacodylate buffer and further with distilled water. En bloc staining was performed using 1% aqueous uranyl acetate and incubated at 4 °C overnight, then rinsed with distilled water. Using the Pelco Biowave, a graded dehydration series (per exchange, 100 Watts for 40 s) was performed using ethanol, transitioned into acetone, and dehydrated specimens were then infiltrated with Epon resin (250 Watt for 3 min) and polymerized at 60 °C overnight. Sections were cut to a thickness of 75 nm using an ultramicrotome (Ultracut UCT, Leica Microsystems, Germany) and a diamond knife (Diatome, Hatfield PA, USA). Images were acquired with a FEI Tecnai F30 transmission electron microscope (FEI, Hillsboro, OR, USA) at 100 kV on a Gatan Ultrascan 4000 CCD (Gatan, Inc., Pleasanton, CA, USA).

### 2.3. Bioassays

Neonate C. includens larvae were infected per os by the droplet feeding method developed by Hughes et al. [21] with five doses of OBs ranging from 1 × 10^4^ to 1 × 10^8^ OBs/mL. Thirty larvae per dose were placed individually on fresh food and monitored two times daily for 8 days. The LC_50_s (concentration of occluded virus required to kill 50% of test larvae) were calculated by PoloPlus as were hypotheses concerning the parallelism and equality of probit dose–response lines (LeOra, Petaluma, CA, USA). Median mortality times (LT_50_s) were calculated with survivors excluded using the Kaplan–Meier Estimator and mortality data of the 1 × 10^6^ OBs/mL dose for both ChinNPV isolates and 1 × 10^7^ OBs/mL dose for AcMNPV. Comparison of LT_50_s was computed using the log-rank test by SigmaPlot version 13 (Systat Software, Inc., San Jose, CA, USA). The bioassay was repeated three times. Droplet feeding assays were also conducted for ChinNPV#1 in *A. gemmatalis*, *Helicoverpa zea*, *Heliothis virescens*, *Spodoptera eridania*, *Spodoptera frugiperda*, and *Trichoplusia ni* neonates (Benzon Research) with doses of OBs ranging from 1 × 10^5^ to 1 × 10^9^ OBs/mL.

### 2.4. Viral DNA Isolation and Sequencing

A 0.75 mL aliquot of ChinNPV#1 at a concentration of 2.23 × 10^9^ OBs/mL was diluted to 28 mL in 0.1 M Na_2_CO_3_. The OBs were solubilized by incubation for 30 min at the benchtop, and the solubilized OBs were neutralized by adding 3.1 mL 1 M Tris-HCl pH 7.6. Insoluble material was removed by centrifugation for 10 min at 1258 *g*, and the supernatant was transferred to a Beckman-Coulter polyallomer ultracentrifuge tube and underlain with 3 mL of 25% *w*/*w* sucrose in phosphate-buffered saline. Occlusion-derived virus (ODV) was pelleted by centrifugation for 75 min at 103,586 *g* using a Beckman SW-28 rotor and L8-8M ultracentrifuge (Beckman Coulter, Brea, CA, USA). DNA was extracted from the ODV pellet and quantified as previously described [22].

A paired-end library was prepared from 100 ng of the DNA sample and sequenced on a MiSeq System (Illumina, Inc., San Diego, CA, USA) as previously described [22]. Sequencing reads were subjected to two rounds of assembly with Lasergene SeqMan NGen 14 (DNASTAR, Inc. Madison, WI, USA). The final genome-length contig was formed from 54,975 reads with an average length of 148 bases and an average coverage of 61.9X. The sequence of the genome was deposited in GenBank with the accession number MK746083.

### 2.5. Genome Feature Identification

Features of the ChinNPV#1 genome sequence to be annotated were identified with the assistance of Lasergene GeneQuest 14 (DNASTAR). Potentially protein-encoding open reading frames (ORFs) of ≥50 codons in size were annotated if they were identified as homologs of other baculovirus ORFs or if they possessed significant sequence similarity to genes from other sources, as determined by BLASTx queries. ORFs with no significant matches to other sequences also were selected for annotation if (a) they did not overlap a larger ORF by ≥75 bp, and (b) they were predicted to be protein-encoding by both the FGENESV (http://linux1.softberry.com/berry.phtml; Softberry Inc., Mount Kisco, NY, USA) and GeneMarkS [23] algorithms. These ORF sequences were also used for HMM-HMM queries with HHpred [24]. A search for regions of conserved sequence repeats in the genome sequence was conducted with Tandem Repeats Finder [25] and the pattern-finding function of Genequest 14.

### 2.6. Sequence Comparison and Phylogeny

For all analyses, nucleotide and amino acid sequences were aligned with MUSCLE [26] as implemented in Lasergene MegAlign Pro 14 (DNASTAR) with default parameters. The classification, names, and GenBank accession numbers of all sequences used in phylogenetic inference are listed in Table S1.

To establish a phylogeny based on baculovirus core gene amino acid alignments, the alignments for the 38 core gene sequences were concatenated using BioEdit 7.2.6 [27]. Maximum likelihood (ML) phylograms were inferred using RAxML [28,29] from the concatenated core gene alignments using the Le and Gascuel (LG) substitution matrix with variable rates among sites, empirical amino acid frequencies, and 100 rapid bootstrap replicates. A core gene phylogeny was also inferred by minimum evolution (ME) as implemented in MEGA X [30] using the Jones–Taylor–Thorton (JTT) substitution matrix with rates varying among sites and a gamma shape parameter value of 0.98. The pairwise-deletion option was used for handling gaps and missing data, and tree reliability was evaluated by bootstrap with 500 replicates.

For phylogenies based on HE65, DNA ligase, and LEF-12 alignments, both ML and ME phylograms were inferred using MEGA X with the JTT substitution matrix with rates varying among sites and a gamma shape parameter value estimated from the alignments. The partial deletion (ML) or pairwise-deletion (ME) option was used for handling gaps and missing data, and tree reliability was evaluated by bootstrap with 500 replicates.

Pairwise Kimura-2-parameter nucleotide distances between ChinNPV#1 and other alphabaculoviruses at the *lef-8*, *lef-9*, and *polh* loci of were determined as previously described [31] using MEGA X. Gene parity plots comparing ORF position and content were constructed as previously descried [32]. Partial alignment of the genome sequences of ChinNPV#1 and related alphabaculoviruses was carried out with Mauve version 20150226 [33].

## 3. Results

### 3.1. Ultrastructural Features of ChinNPV#1 OBs

**Figure 1:**
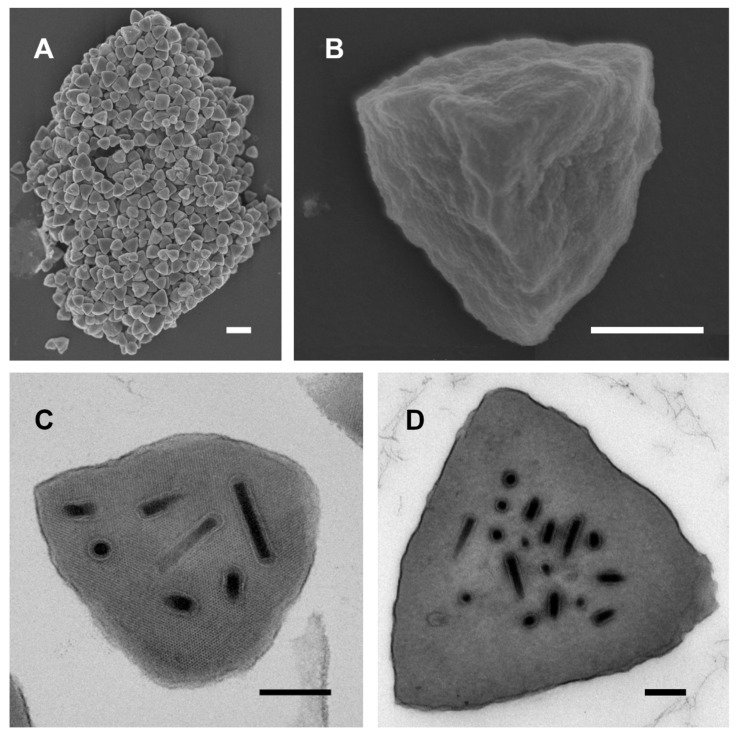
Scanning and transmission electron micrographs of ChinNPV#1 occlusion bodies (OBs): (**A**) A group of ChinNPV#1 OBs; (**B**) high-magnification view of a single OB; (**C**) section through a single OB, showing the lattice lines of the paracrystalline polyhedrin matrix surrounding the occluded virions; (**D**) section through a larger OB. Scale bars: (**A**) 2 µm, (**B**) 500 nm, (**C**) and (**D**) 200 nm.

Electron microscopy revealed that ChinNPV#1 OBs possess a distinctive tetrahedral shape (Figure 1A,B) reminiscent of the OBs produced by Thysanoplusia orichalcea single nucleopolyhedrovirus (ThorSNPV), an alphabaculovirus isolated from Indonesian specimens of *Thysanoplusia orichalcea* [34]. The ChinNPV#1 OBs ranged in size from 0.82 to 1.62 µm per side, and contained virions consisting of singly-enveloped nucleocapsids measuring 230 × 27 nm (Figure 1C,D).

### 3.2. Pathogenicity of ChinNPV#1

Larvae infected with ChinNPV#1 diplayed mortality with hallmark symptoms of nuclear polyhedrosis, including cuticular fragility and tissue liquefaction. In lethal concentration bioassays ChinNPV#1 killed neonate *C. includens* larvae with LC_50_ values in a similar range as ChinNPV-460, 1.7–1.9 × 10^5^ OBs/mL (Table 1). AcMNPV was 10× less infectious than both of the ChinNPVs. In lethal time bioassays, both ChinNPV-460 and AcMNPV killed larvae approximately twice as fast as ChinNPV#1 (Table 1). In futher testing, the host range of ChinNPV#1 was found to be very narrow and was non-infectious to *A. gemmatalis*, *H. zea*, *H. virescens*, *S. eridania*, *S. frugiperda*, and *T. ni* neonate larvae.

### 3.3. Characteristics of the ChinNPV#1 Genome Sequence

**Figure 2:**
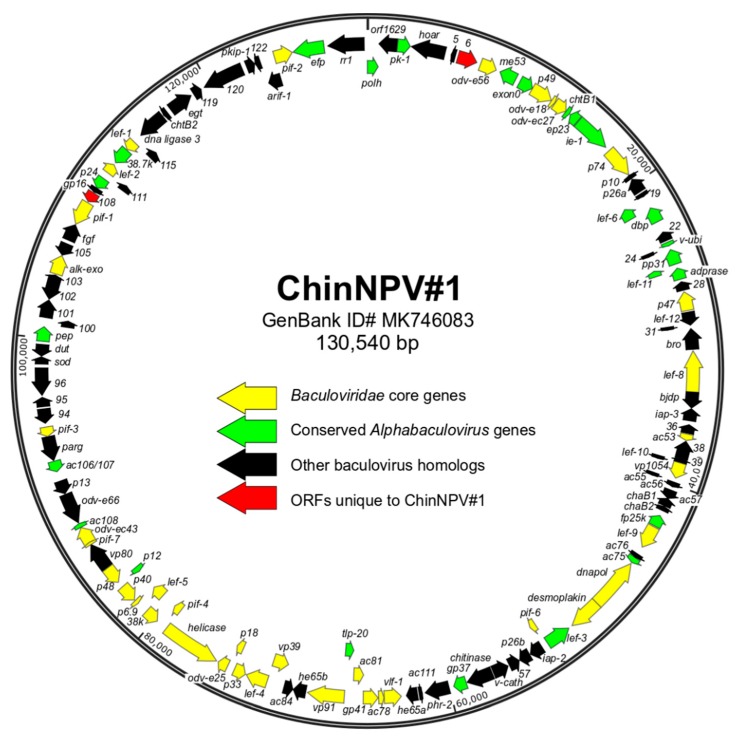
Open reading frame (ORF) map of the ChinNPV#1 genome. ORFs are represented by arrows, with the position and direction of the arrow indicating ORF position and orientation. Each ORF is color-coded to indicate whether it corresponds to a baculovirus core gene (yellow), an ORF reported to be conserved among all alphabaculoviruses (green) [36], an ORF with homologs in a subset of other baculoviruses (black), or an ORF not previously identified in any other baculovirus genome (red). ORFs are designated by either a specific name, the designation of their AcMNPV homolog (*acXX*), or a number corresponding to its annotation in the ChinNPV#1 genome.

Sequencing of ChinNPV#1 DNA revealed the consensus viral genome sequence to be a circular molecule of 130,540 bp with a nucleotide distribution of 37.28% C + G and a set of 126 ORFs identified for annotation (Figure 2, Table S2). However, examination of the genome sequence did not reveal any homologous repeat (*hr*) regions, which are regions of conserved direct repeats and/or palindromes dispersed throughout the genomes of most other baculoviruses [35]. Instead, three intergenic regions were found to contain direct repeats of three distinct non-conserved sequences: (a) one partial and five complete perfect copies of the sequence 5′-TGTATTAGCTTTAATCTATTAT-3′ at nt 17081-17204; (b) four copies of the consensus sequence 5′-AAAAARTATTATAAACATTTCAAAG-3′ at nt 51336-51437; and (c) two perfect copies of the sequence 5′-TACAGTTAACAAAAAACCCAATCACCATTAGAA-3′ at nt 96248-96313. Sequences with significant similarity to these repeats were not found to occur in related alphabaculoviruses.

A total of 16,491 putative single-nucleotide polymorphisms (SNPs) and insertions/deletions (indels) were identified among the sequencing reads with the SNP Report function of Lasergene SeqMan Pro 14 (DNASTAR). These variants ranged in frequency from 0.95% to 13.63% of reads at variant positions, but only 62 of the variants were present at frequencies higher than 5%, suggesting that the ChinNPV#1 isolate is genetically relatively homogenous.

### 3.4. Relationship of ChinNPV#1 to Other Baculoviruses

**Figure 3:**
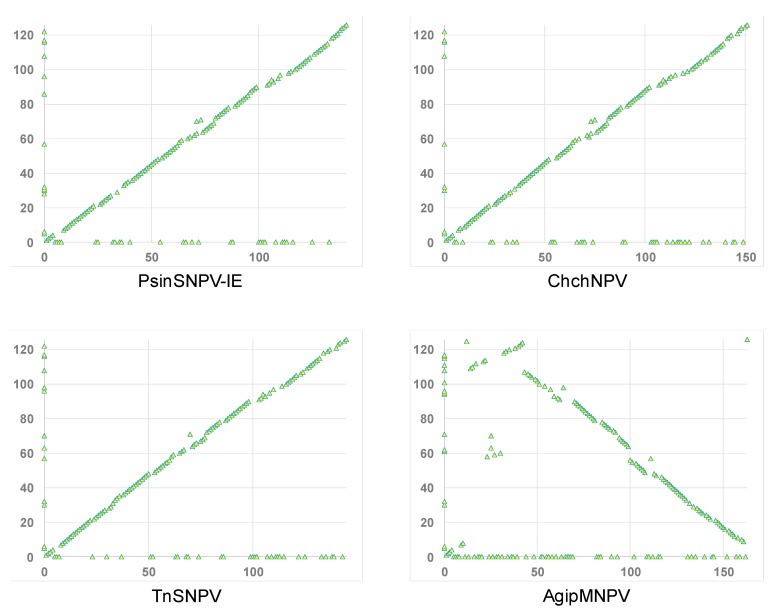
Gene parity plots comparing the ORF content and order of ChinNPV#1 (*y*-axis) with that of related alphabaculoviruses from noctuid hosts of subfamilies Plusiinae (PsinSNPV-IE, ChchNPV, and TnSNPV) and Noctuinae (AgipMNPV). Each point in a plot represents an ORF, and points corresponding to ORFs present in only one of the compared genomes appear on the axis line for the virus in which they are present.

BLASTx queries with ChinNPV#1 ORF sequences indicated that ChinNPV#1 was closely related to other Group II alphabaculoviruses from moths of the noctuid subfamily Plusiinae, including PsinSNPV-IE [18], Chrysodeixis chalcites nucleopolyhedrovirus (ChchNPV; [37]), and Trichoplusia ni single nucleopolyhedrovirus (TnSNPV; [38]). These results are consistent with those of gene parity plot analysis, which indicated that the content and order of homologous ORFs among the Group II plusiine alphabaculoviruses was relatively well-conserved compared to a representative virus (Agrotis ipsilon multiple nucleopolyhedrovirus, AgipMNPV) of a clade of alphabaculoviruses from subfamily Noctuinae (Figure 3). The PsinSNPV-IE, ChchNPV, and TnSNPV genomes also lack hr regions, indicating that the absence of this feature is a trait of the alphabaculoviruses in this clade.

**Figure 4:**
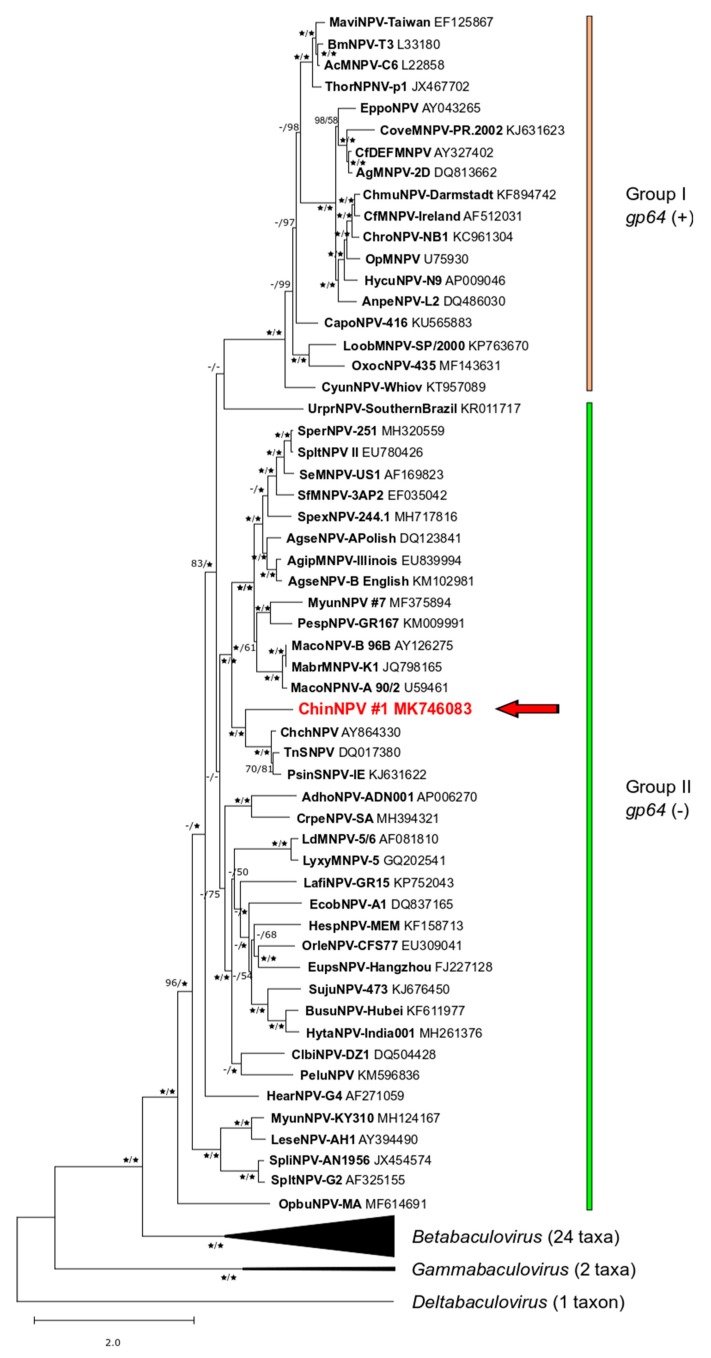
Relationships among ChinNPV#1, representative isolates of other baculovirus species, and other unclassified baculovirus isolates inferred from the predicted amino acid sequences of baculovirus core genes. A phylogram was constructed from the concatenated alignments of 38 baculovirus core gene amino acid sequences using maximum likelihood (ML) and minimum evolution (ME) methods. Shown is the ML tree with bootstrap values >50% for branches in trees produced by both ME and ML methods (displayed as ME/ML). Stars indicate bootstrap values of 100%. Branches for the viruses of genera *Betabaculovirus*, *Gammabaculovirus*, and *Deltabaculovirus* are collapsed, and the numbers of taxa in those nodes are shown in parentheses. Group I and Group II alphabaculoviruses are indicated with colored bars. ChinNPV#1 is indicated in bold red type, and its position denoted with a red arrow. The taxa and sequences used in the analysis are as listed in Table S1.

Phylogenetic inference based on concatenated core gene amino acid sequences placed ChinNPV#1 in a well-supported clade with the above-mentioned virus isolates. This plusiine alphabaculovirus clade in turn is part of Clade II.a, a well-defined clade of alphabaculoviruses from hosts of the lepidopteran superfamily Noctuoidea [39]. ChinNPV#1 occupies a basal position relative to PsinSNPV-IE, ChchNPV, and TnSNPV, indicating that it diverged from these viruses relatively early, even though it shares a host species of origin with PsinSNPV-IE (Figure 4).

The ranges for Kimura-2-parameter nucleotide distances separating ChinNPV#1 from ChchNPV, PsinNPV-IE, and TnSNPV at the *lef-8*, *lef-9*, and *polh* loci are 1.01–1.02, 0.44–0.51, and 0.17–0.19 substitutions/site, respectively, indicating that ChinNPV#1 cannot be classified as a member of the currently existing species *Chrysodeixis chalcites nucleopolyhedrovirus*, *Chrysodeixis includens nucleopolyhedrovirus*, or *Trichoplusia ni single nucleopolyhedrovirus* [31].

### 3.5. ORF content of ChinNPV#1

#### 3.5.1. Core Genes, Gene Families, and ORFs Missing from/Unique to ChinNPV#1

The ChinNPV#1 genome contains all 38 baculovirus core genes, including *pif-7* (*ac110*) [36,40]. There is no *pif-7* ORF annotated in the PsinSNPV-IE genome, but a 31-codon ORF present at nt 92,069→92,161 of the PsinSNPV-IE sequence encodes an amino acid sequence with 96.6% identity to residues 32–60 of the ChchNPV PIF-7 sequence. Annotations for none of the other ChinNPV isolates sequenced (IA-ID, IF, and IG) include a record for *pif-7*, but examination of the sequences for these isolates revealed the presence of full-length *pif-7* homologs of 62 or 64 codons in each of these genomes.

A single member of the *bro* (*baculovirus repeated ORF*) gene family was identified in the ChinNPV#1 sequence just upstream of the *lef-8* gene. This ORF (ORF32) encodes a 441-amino acid sequence that does not appear to be an ortholog of any of the BRO sequences encoded in the PsinSNPV-IE, ChchNPV, or TnSNPV genomes. The ChinNPV#1 sequence also contains a duplicate copy of the *p26* gene, as reported for several other group II alphabaculoviruses [18].

A number of ORFs present in the other plusiine Clade II.a alphabaculoviruses are missing from ChinNPV#1, including homologs of AcMNPV ORFs *ac26*, *ac49* (*pcna*), and *ac63*. Also missing are homologs of *protein tyrosine phosphatase-2* (*ptp-2*) and *rr2*, which encodes the small subunit of ribonucleotide reductase. The absence of the latter homolog from ChinNPV#1 is noteworthy, because ChinNPV#1 encodes a homolog of the large subunit of ribonucleotide reductase (*rr1*). Almost all baculoviruses that carry *rr1* also encode *rr2*, with the sole exception being isolates of Spodoptera frugiperda multiple nucleopolyhedrovirus [41,42].

Two ORFs, ORF6 and ORF108, were identified that were not represented by homologs in other baculovirus genomes. ORF6 and ORF108 encode 419- and 216-amino acid sequences, respectively, with no identifiable motifs or significant sequence similarity to other sequences identifiable in BLASTx or hhPRED queries. One copy each of an initiator motif (CAKT) and a TATA box (TATAWAW) are present within 250 bp of the start codons of these ORFs.

#### 3.5.2. Polyhedrin

**Figure 5:**
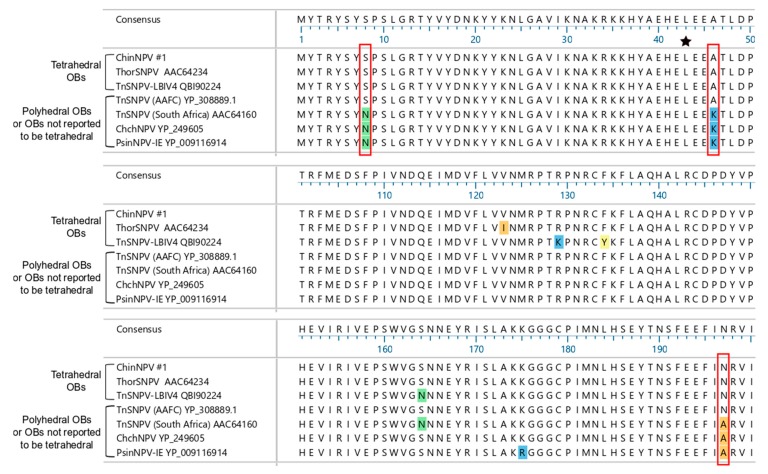
Alignment of Group II plusiine alphabaculovirus polyhedrin amino acid sequences. Portions of a MUSCLE alignment of these sequences where differences among the sequences exist are shown, encompassing residues 1–50, 101–150, and 151–200, along with the consensus sequence in those regions. Amino acid identities that differ from the consensus identity at a given position are highlighted in a color corresponding to the biochemical class of the residue. Residues that are conserved among alphabaculoviruses with tetrahedral OBs and that differ in the three of the four sequences of alphabaculoviruses not reported to produce tetrahedral OBs are outlined in red. A star indicates a residue (#43) previously reported to determine tetrahedral OB morphology [47]. TnSNPV (AAFC) corresponds to the exemplar TnSNPV isolate whose genome was reported in reference [38]; the other viruses are as described in the text.

In addition to ChinNPV#1, ThorSNPV [34] and the TnSNPV isolate LBIV-4 [43] have been reported to produce OBs with a tetrahedral morphology. ChchNPV and PsinSNPV-IE were shown to produce OBs of a more typical polyhedral morphology [12,44], while the exemplar isolate of TnSNPV [38] and an isolate of TnSNPV from South Africa [45] were not specifically reported to produce tetrahedral OBs. To identify individual polyhedrin amino acid residues associated with a tetrahedral OB morphology, the ChinNPV#1 polyhedrin sequence was aligned with the polyhedrin sequences of ThorSNPV, ChchNPV, PsinSNPV-IE, and three different isolates of TnSNPV for which a complete polyhedrin sequence had been determined. The identities of residues 8, 46, and 197 were found to be conserved among the polyhedrins of tetrahedral OBs (Figure 5). These positions were occupied by Ser, Ala, and Asn, respectively, in the polyhedrin sequences of ChinNPV#1, ThorSNPV, and TnSNPV-LBIV-4. The same three positions were occupied by different amino acids—Asn, Lys, and Ala, respectively—in the polyhedrins of three of the remaining viruses, ChchNPV, PsinSNPV-IE, and the South African isolate of TnSNPV, suggesting that amino acid identities at these positions may influence or determine OB morphology. However, the polyhedrin sequence of the TnSNPV exemplar isolate [38], which was not reported to produce tetrahedral OBs, was 100% identical to the sequence of ChinNPV#1 polyhedrin, indicating that the correlation between the residue encoded at these positions and plusiine alphabaculovirus OB morphology was not perfectly positive.

An alphabaculovirus from *Spilarctia obliqua* (lepidopteran family Arctiidae; SpobNPV) also produces tetrahedral OBs [46]. The partial polyhedrin sequence of SpobNPV aligned with ChinNPV#1 polyhedrin over a region extending from residues 62-222, and also encoded an Asn at position 197. However, the SpobNPV sequence only shared 88.8% sequence identity overall with that of ChinNPV#1 polyhedrin.

#### 3.5.3. *Lef-12*

**Figure 6:**
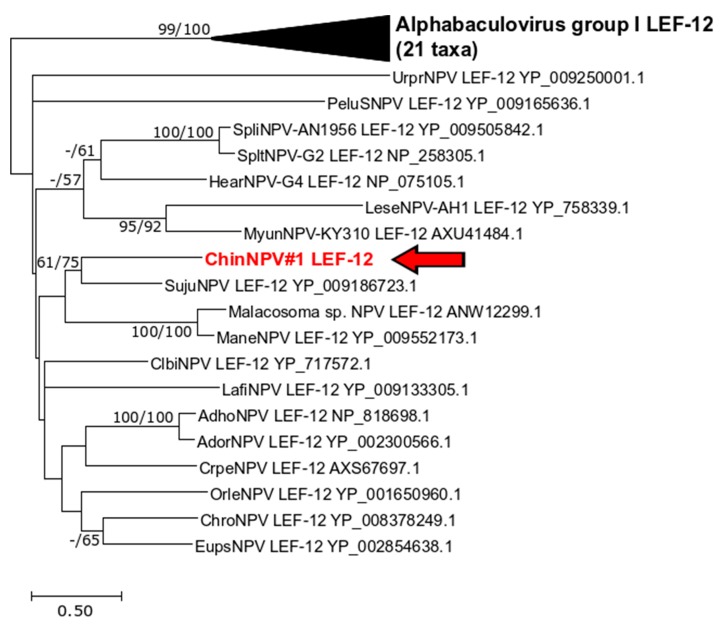
Phylogenetic inference of relationships among encoded baculovirus LEF-12 proteins. An ML phylogram shown with branch support for ME and ML phylogenies as described in the legend for Figure 4. The position of the ChinNPV#1 sequence is indicated in bold red type with an arrow. Branches for the 21 Group I alphabaculovirus sequences are collapsed.

ORF30 of ChinNPV#1 exhibited significant sequence similarity with *lef-12* (AcMNPV ORF *ac41*), a gene originally identified in a screen for baculovirus genes required for late-phase gene expression [48,49] and found in several alphabaculovirus genomes. ChchNPV, PsinNPV-IE, and TnSNPV all lack a *lef-12* homolog, and the closest match to ORF30 was with the *lef-12* homolog of Sucra jujuba nucleopolyhedrovirus [50]. Phylogenetic inference from a LEF-12 amino acid sequence alignment placed ORF30 with the SujuNPV LEF-12 with reasonably good bootstrap support in both the ML and ME trees (Figure 6). In general, the Group II alphabaculovirus LEF-12 sequences were relatively divergent, and bootstrap support that was >50% existed mostly for the terminal branches in the phylogram. The phylogeny suggests that ChinNPV#1 may have obtained its copy of *lef-12* from a non-plusiine alphabaculovirus, although the possibility that the other Group II plusiine alphabaculovirus lineages also possessed a *lef-12* gene at one point but subsequently lost the sequence cannot be excluded.

#### 3.5.4. Two Full-Length Copies of *he65*

**Figure 7:**
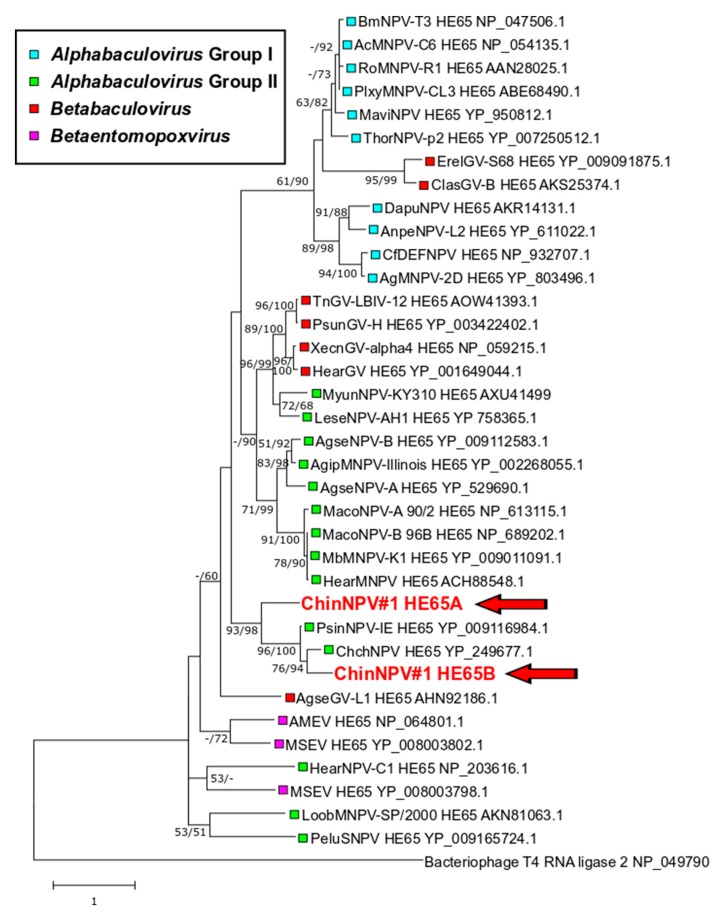
Phylogenetic inference of relationships among encoded baculovirus HE65 amino acid sequences. An ML phylogram inferred with a bacteriophage T4 RNA ligase included as an outgroup is shown with branch support for ME and ML phylogenies as described in the legend for Figure 4. The position of the two ChinNPV#1 HE65 sequences are indicated in bold red type with arrows. Genus and group classifications of the different HE65 taxa are indicated with colored boxes.

Homologs of the *he65* gene (AcMNPV ORF *ac105*), transcribed early during infection [51], have been found in a variety of alpha- and betabaculoviruses and entomopoxviruses. An hhPRED query with a MUSCLE alignment of HE65 sequences produced matches with the *RNA_lig_T4_1* (PF09511; probability = 98.88%) and *RNA_ligase* (PF09414; probability = 97.75%) protein families, which are RNA ligases involved in a variety of different functions. The ChinNPV#1 genome was found to contain two complete *he65* homologs, ORF63 (*he65a*) and ORF70 (*he65b*). Phylogenetic inference with an alignment of HE65 amino acid sequences placed both HE65A and HE65B in a clade with the HE65 sequences of ChchNPV and PsinSNPV-IE. Both phylogeny and sequence similarity indicated that *he65b* is more closely related to the *he65* ORFs of ChchNPV and PsinSNPV-IE. HE65A occupied a basal position in the HE65 clade of these viruses, suggesting that an intragenomic recombination event resulted in a duplication of *he65* and that the two *he65* ORFs have been under different selection pressures since the duplication event. From the Figure 7 tree, the distribution of *he65* ORFs appears to be characterized by horizontal gene transfer events, especially between alpha- and betabaculoviruses.

**Figure 8:**
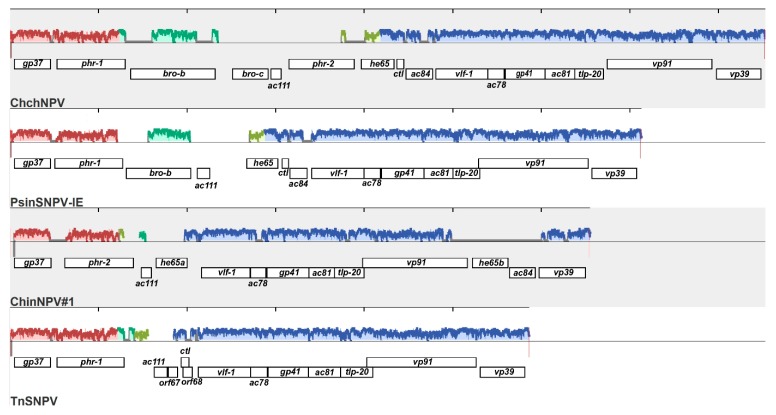
Mauve alignment of the genomic region of ChinNPV#1 and three other plusiine alphabaculoviruses extending from *gp37* to *vp39*, indicating the positions of homologous ORFs in these viruses. Outlines of the same color correspond to Locally Collinear Blocks (LCBs) of sequence that are conserved among the viruses. The height of the profile within each LCB corresponds to the average level of sequence conservation among the isolates in that region of the genome sequence. ORFs annotated for each virus are indicated below the LCBs.

A Mauve alignment of the regions containing *he65* from ChchNPV, PsinSNPV-IE, ChinNPV#1, and TnSNPV indicated that this region has been subjected to a significant degree of rearrangement during the diversification of this lineage of viruses. In ChchNPV and PsinSNPV-IE, the *vp39* and *vp91* ORFs are directly adjacent to each other, while *he65*, *ctl*, and *ac84* ORFs are located upstream of *vlf-1*. In ChinNPV#1, *he65b* and *ac84* are located between *vp39* and *vp91*, and no *ctl* ORF is present. ChchNPV has two DNA photolyase homologs, *phr-1* and *phr-2*, which are separated by two *bro* sequences (*bro-b* and *bro-c*) and a homolog of AcMNPV *ac111*. In contrast, PsinSNPV-IE only has homologs of *phr-1*, *bro-b*, and *ac111*. ChinNPV#1 has only a homolog of *phr-2*, and it is located downstream of *gp37* in the position where *phr-1* is found in the other three viruses. The *he65*, *ac84*, and *phr-2* ORFs are missing from TnSNPV, but the identification and distribution of locally collinear blocks from the Mauve analysis suggests that portions of these sequences may be present in TnSNPV (Figure 8).

#### 3.5.5. DNA Ligase 3

**Figure 9:**
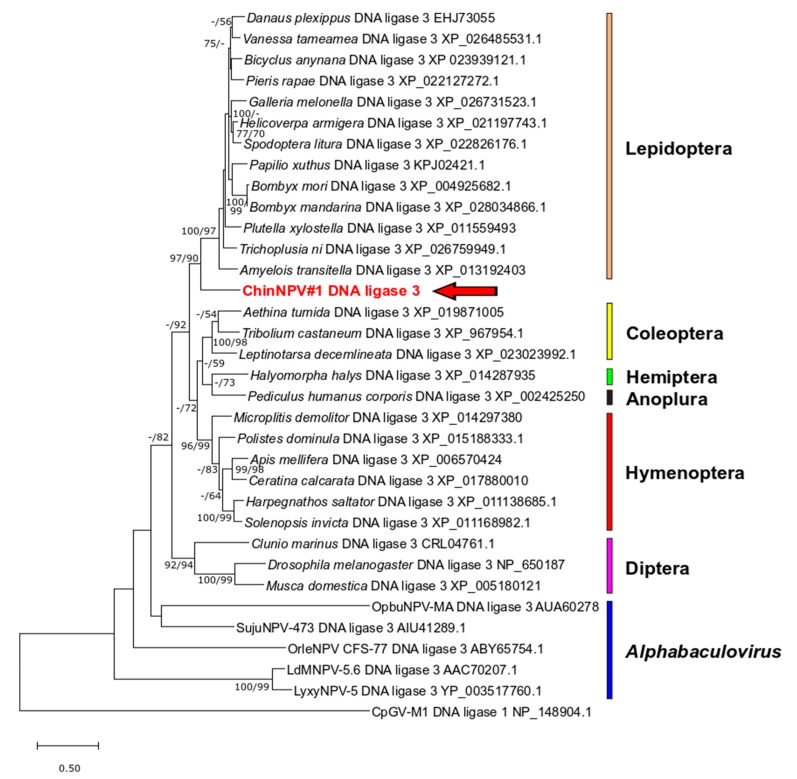
Phylogenetic inference of relationships among insect and alphabaculovirus DNA ligase 3 (LIG3) amino acid sequences. An ML phylogram inferred with a betabaculovirus DNA ligase 1 deployed as an outgroup is shown with branch support for ME and ML phylogenies as described in the legend for Figure 4. Insect order classifications are indicated with colored bars for different clades and taxa of LIG3 sequences, as are the positions of the alphabaculovirus LIG3 sequences. The position of the ChinNPV#1 LIG3 sequence is indicated in bold red type with an arrow.

ChinNPV#1 ORF116 encoded an amino acid sequence which exhibited significant sequence similarity with DNA ligase 3 (LIG3) sequences from insect sources in BLASTx queries. LIG3 homologs have also been identified in a small number of other alphabaculovirus genomes. Phylogenetic inference based on an alignment of ORF116 and other alphabaculovirus and selected insect LIG3 sequences grouped the ChinNPV#1 LIG3 sequence with sequences from other lepidopterans with strong bootstrap support in both ML and ME trees (97/90; Figure 9). In contrast, the other baculovirus LIG3 sequences were not grouped together and were separated by long branches.

## 4. Discussion

Clade II.a is one of the better-defined clades among the paraphyletic assembly of Group II alphabaculoviruses [39]. Most of the fully-sequenced alphabaculoviruses in this clade originate from hosts of lepidopteran subfamily Noctuinae [52], but ChinNPV#1 belongs to the group of Clade II.a viruses from subfamily Plusiinae. Two members of this group, ChinNPV#1 and PsinSNPV-IE, both originate from the *C. includens*, and the host range of ChinNPV#1 appears to be confined to this species (see Section 3.2). This is consistent with the observation that, though baculoviruses often possess a narrow host range consisting of one or a few related species, larvae of a single lepidopteran species can be susceptible to multiple different baculoviruses [53,54,55,56].

Polyhedrin sequence phylogeny indicates that ThorSNPV is also a part of this clade [34,57]. Although ChinNPV#1, ThorSNPV, and one isolate of TnSNPV are known to produce tetrahedral OBs, OB morphology doesn’t appear to be a reliable taxonomic character for plusiine alphabaculoviruses. When the polyhedrin sequence of AcMNPV was replaced with that of ThorSNPV, tetrahedral OBs containing the multiple-nucleocapsid ODV of AcMNPV were produced [47]. Site-directed mutagenesis to change the Ile encoded at position 43 of the AcMNPV polyhedrin sequence to Leu, the residue present at this position in the ThorSNPV sequence, also resulted in tetrahedral OBs [47]. However, Leu is encoded at this position in every Clade II.a polyhedrin sequence, including those that are assembled in polyhedral OBs. This observation indicates that the results of the Ile to Leu substitution are likely restricted to the AcMNPV and similar polyhedrin sequences.

The ChinNPV#1 genome was distinguished from the other Clade II.a looper alphabaculovirus genomes not only by sequence divergence, but by ORF content. While the ChinNPV#1 *lef-12* homolog was clearly from another baculovirus, it is unclear if ChinNPV#1 acquired a copy of *lef-12* by recent horizontal gene transfer from another Group II alphabaculovirus or if the other Clade II.a looper viruses lost a *lef-12* gene that had been present in an ancestral looper alphabaculovirus prior to the diversification of the lineage. The significance of the presence of *lef-12* in ChinNPV#1 is unknown, since a subsequent study on *lef-12* knockout mutants of AcMNPV indicated that *lef-12* was not required for late gene expression or replication [58]. The DNA ligase 3 homolog of ChinNPV#1, in contrast, appears to have been a recent acquisition from a lepidopteran host. A LIG3 homolog encoded by Lymantria dispar multiple nucleopolyhedrovirus possessed the ability to ligate DNA substrates, and Rohrmann [9] has proposed that this enzyme may work in concert with a second baculovirus-encoded DNA helicase (*helicase-2*) to process Okazaki fragments generated during lagging-strand DNA synthesis. Although homologs of *helicase-2* are present in five of the six other alphabaculoviruses that encode a LIG3 sequence, no *helicase-2* homologs were identified in the ChinNPV#1 genome. The functional significance of the LIG3 ORF in ChinNPV#1 is thus unclear.

An analysis of the *he65* homologs in ChinNPV#1 provides an example of how genomic rearrangement accompanied by gene loss and acquisition can contribute to genetic divergence of related baculoviruses. While the *he65* homologues of Choristoneura fumiferana DEF multiple nucleopolyhedrovirus and some Anticarsia gemmatalis multiple nucleopolyhedrovirus isolates were found to be split into two discrete ORFs [59,60,61], the occurrence of two full-length *he65* ORFs has not been previously reported in a baculovirus genome. The phylogenies of *he65* in this study and by Harrison and co-workers [56] indicate that this ORF has been particularly mobile among different taxa of insect viruses. It is unclear if there is any selective advantage to having two copies of *he65* present in the genome.

As a novel alphabaculovirus from the soybean looper, ChinNPV#1 is another potential tool for control of this pest in the event that *C. includens* populations develop resistance to other soybean looper baculoviruses. Although it has a relatively slow speed of kill, this property does not necessarily indicate that larvae infected with this virus will consume more soybean foliage—ChinNPV#1-infected larvae may undergo a prolonged moribund phase prior to dying, as has been observed with larvae of *Agrotis ipsilon* infected with its native alphabaculovirus [62]. In addition, the data from its genome sequence contributes to our knowledge of baculovirus genetics and evolution. Further studies of this virus and its genes will advance our understanding of baculovirus features such as OB morphology.

## Figures and Tables

**Table 1:** Dose-mortality (LC_50_) and time-mortality (LT_50_) response of neonate C. includens infected with ChinNPV and AcMNPV.

		Fiducial Limits			Median		
Virus	LC_50_^a^ (OBs/mL)	Lower Limit	Upper Limit	Slope	LT_50_^ab^ (hr p.i.)	95% CL	% Mortality
ChinNPV#1	1.71 × 10^5^ **a**	9.97 × 10^4^	2.90 × 10^5^	1.8 ± 0.3	166.9 **a**	164.5–169.3	76.7
ChinNPV-460	1.87 × 10^5^ **a**	9.00 × 10^4^	3.43 × 10^5^	1.5 ± 0.3	91.3 **b**	61.2–121.4	86.7
AcMNPV	1.40 × 10^6^ **b**	8.46 × 10^5^	2.31 × 10^6^	1.7 ± 0.3	77.6 **b**	75.3–79.9	93.3

**a** Values with different letters are significantly different at *p* < 0.05; **b** Median lethal time of insects infected was determined by the Kaplan–Meier estimator and reported with 95% confidence limits. hr p. i., hours post infection.

## Supplementary Materials

The following are available online at https://www.mdpi.com/1999-4915/11/7/579/s1, Table S1: Names, abbreviations, and GenBank accession numbers of taxa used in phylogenetic inference, Table S2: ChinNPV#1 ORFs.

## References

[1] Eichlin T.D., Cunningham H.B. (1978). The Plusiinae (Lepidoptera: Noctuidae) of America north of Mexico, Emphasizing Genitalic and Larval Morphology.

[2] Kogan M., Turnipseed S.G. (1987). Ecology and management of soybean arthropods. Ann. Rev. Entomol..

[3] Bortolotto O.C., de F. Bueno R.C.O., de F. Bueno A., da Kruz A.K.S., Queiroz A.P., Sanzovo A., Ferreira R.B. (2015). The use of soybean integrated pest management in Brazil: A review. Agron. Sci. Biotechnol..

[4] Mascarenhas R.N., Boethel D.J. (1997). Responses of field-collected strains of soybean looper (Lepidoptera:Noctuidae) to selected insecticides using an artificial diet overlay bioassay. J. Econ. Entomol..

[5] Owen L.N., Catchot A.L., Musser F.R., Gore J., Cook D.C., Jackson R. (2013). Susceptibility of *Chrysodeixis includens* (Lepidoptera:Noctuidae) to reduced-risk insecticides. Fla. Entomol..

[6] Bueno R.C.O.D., Parra J.R.P., Bueno A.D. (2012). *Trichogramma pretiosum* parasitism of *Pseudoplusia includens* and *Anticarsia gemmatalis* eggs at different temperatures. Biol. Control.

[7] Lacey L.A., Grzywacz D., Shapiro-Ilan D.I., Frutos R., Brownbridge M., Goettel M.S. (2015). Insect pathogens as biological control agents: Back to the future. J. Invertebr. Pathol..

[8] Haase S., Sciocco-Cap A., Romanowski V. (2015). Baculovirus insecticides in Latin America: Historical overview, current status and future perspectives. Viruses.

[9] Rohrmann G.F. (2013). Baculovirus Molecular Biology.

[10] Harrison R.L., Herniou E.A., Jehle J.A., Theilmann D.A., Burand J.P., Becnel J.J., Krell P.J., van Oers M.M., Mowery J.D., Bauchan G.R. (2018). ICTV Virus Taxonomy Profile: Baculoviridae. J. Gen. Virol..

[11] Livingston J.M., Yearian W.C. (1972). Nuclear polyhedrosis virus of *Pseudoplusia includens* (Lepidoptera-Noctuidae). J. Invert. Path..

[12] Alexandre T.M., Ribeiro Z.M., Craveiro S.R., Cunha F., Fonseca I.C., Moscardi F., Castro M.E. (2010). Evaluation of seven viral isolates as potential biocontrol agents against *Pseudoplusia includens* (Lepidoptera: Noctuidae) caterpillars. J. Invertebr. Pathol..

[13] Livingston J.M., Mcleod P.J., Yearian W.C., Young S.Y. (1980). Laboratory and field evaluation of a nuclear polyhedrosis virus of the soybean looper, *Pseudoplusia includens*. J. Georgia Entomol. So..

[14] Mcleod P.J., Young S.Y., Yearian W.C. (1982). Application of a baculovirus of *Pseudoplusia includens* to soybean—efficacy and seasonal persistence (Lepidoptera, Noctuidae). Environ. Entomol..

[15] Muraro D.S., Giacomelli T., Stacke R.F., Godoy D.N., Marcon P., Popham H.J.R., Bernardi O. (2019). Baseline susceptibility of Brazilian populations of *Chrysodeixis includens* (Lepidoptera: Noctuidae) to C. includens nucleopolyhedrovirus and diagnostic concentration for resistance monitoring. J. Econ. Entomol..

[16] Godoy D.N., Führ F.M., Stacke R.F., Muraro D.S., Marçon P., Popham H.J.R., Bernardi O. (2019). No cross-resistance between ChinNPV and chemical insecticides in *Chrysodeixis includens* (Lepidoptera: Noctuidae). J. Invert. Path..

[17] Craveiro S.R., Melo F.L., Ribeiro Z.M., Ribeiro B.M., Bao S.N., Inglis P.W., Castro M.E. (2013). Pseudoplusia includens single nucleopolyhedrovirus: Genetic diversity, phylogeny and hypervariability of the pif-2 gene. J. Invertebr. Pathol..

[18] Craveiro S.R., Inglis P.W., Togawa R.C., Grynberg P., Melo F.L., Ribeiro Z.M., Ribeiro B.M., Bao S.N., Castro M.E. (2015). The genome sequence of Pseudoplusia includens single nucleopolyhedrovirus and an analysis of p26 gene evolution in the baculoviruses. BMC Genom..

[19] Craveiro S.R., Santos L.A.V.M., Togawa R.C., Inglis P.W., Grynberg P., Ribeiro Z.M.A., Ribeiro B.M., Castro M.E.B. (2016). Complete genome sequences of six Chrysodeixis includens nucleopolyhedrovirus isolates from Brazil and Guatemala. Microbiol. Resour. Ann..

[20] Adams M.J., Lefkowitz E.J., King A.M., Harrach B., Harrison R.L., Knowles N.J., Kropinski A.M., Krupovic M., Kuhn J.H., Mushegian A.R. (2016). Ratification vote on taxonomic proposals to the International Committee on Taxonomy of Viruses (2016). Arch. Virol..

[21] Hughes P.R., van Beek N.A.M., Wood H.A. (1986). A modified droplet feeding method for rapid assay of *Bacillus thuringiensis* and baculoviruses in noctuid larvae. J. Invert. Path..

[22] Harrison R.L., Rowley D.L., Mowery J.D., Bauchan G.R., Burand J.P. (2017). The Operophtera brumata nucleopolyhedrovirus (OpbuNPV) represents an early, divergent lineage within genus *Alphabaculovirus*. Viruses.

[23] Besemer J., Lomsadze A., Borodovsky M. (2001). GeneMarkS: A self-training method for prediction of gene starts in microbial genomes. Implications for finding sequence motifs in regulatory regions. Nuc. Acids. Res..

[24] Zimmermann L., Stephens A., Nam S.Z., Rau D., Kubler J., Lozajic M., Gabler F., Soding J., Lupas A.N., Alva V. (2018). A completely reimplemented MPI bioinformatics toolkit with a new HHpred server at its core. J. Mol. Biol..

[25] Benson G. (1999). Tandem repeats finder: A program to analyze DNA sequences. Nuc. Acids. Res..

[26] Edgar R.C. (2004). MUSCLE: A multiple sequence alignment method with reduced time and space complexity. BMC Bioinform..

[27] Hall T.A. (1999). BioEdit: A user-friendly biological sequence alignment editor and analysis program for Windows 95/98/NT. Nucleic Acids Symp. Ser..

[28] Silvestro D., Michalak I. (2012). raxmlGUI: A graphical front-end for RAxML. Org. Divers. Evol..

[29] Stamatakis A. (2014). RAxML version 8: A tool for phylogenetic analysis and post-analysis of large phylogenies. Bioinformatics.

[30] Kumar S., Stecher G., Li M., Knyaz C., Tamura K. (2018). MEGA X: Molecular Evolutionary Genetics Analysis across computing platforms. Mol. Biol. Evol..

[31] Jehle J.A., Lange M., Wang H., Hu Z., Wang Y., Hauschild R. (2006). Molecular identification and phylogenetic analysis of baculoviruses from Lepidoptera. Virology.

[32] Hu Z.H., Arif B.M., Jin F., Martens J.W., Chen X.W., Sun J.S., Zuidema D., Goldbach R.W., Vlak J.M. (1998). Distinct gene arrangement in the Buzura suppressaria single-nucleocapsid nucleopolyhedrovirus genome. J. Gen. Virol..

[33] Darling A.E., Mau B., Perna N.T. (2010). progressiveMauve: Multiple genome alignment with gene gain, loss and rearrangement. PLoS ONE.

[34] Cheng X.W., Carner G.R. (2000). Characterization of a single-nucleocapsid nucleopolyhedrovirus of *Thysanoplusia orichalcea* L. (Lepidoptera: Noctuidae) from Indonesia. J. Invert. Path..

[35] Van Oers M.M., Vlak J.M. (2007). Baculovirus genomics. Curr. Drug Targets.

[36] Garavaglia M.J., Miele S.A., Iserte J.A., Belaich M.N., Ghiringhelli P.D. (2012). The *ac53*, *ac78*, *ac101*, and *ac103* genes are newly discovered core genes in the family *Baculoviridae*. J. Virol..

[37] van Oers M.M., Abma-Henkens M.H., Herniou E.A., de Groot J.C., Peters S., Vlak J.M. (2005). Genome sequence of Chrysodeixis chalcites nucleopolyhedrovirus, a baculovirus with two DNA photolyase genes. J. Gen. Virol..

[38] Willis L.G., Seipp R., Stewart T.M., Erlandson M.A., Theilmann D.A. (2005). Sequence analysis of the complete genome of Trichoplusia ni single nucleopolyhedrovirus and the identification of a baculoviral photolyase gene. Virology.

[39] Theze J., Lopez-Vaamonde C., Cory J.S., Herniou E.A. (2018). Biodiversity, evolution and ecological specialization of baculoviruses: A treasure trove for future applied research. Viruses.

[40] Javed M.A., Biswas S., Willis L.G., Harris S., Pritchard C., van Oers M.M., Donly B.C., Erlandson M.A., Hegedus D.D., Theilmann D.A. (2017). Autographa californica multiple nucleopolyhedrovirus AC83 is a per os infectivity factor (PIF) protein required for occlusion-derived virus (ODV) and budded virus nucleocapsid assembly as well as assembly of the PIF complex in ODV envelopes. J. Virol..

[41] Harrison R.L., Puttler B., Popham H.J. (2008). Genomic sequence analysis of a fast-killing isolate of Spodoptera frugiperda multiple nucleopolyhedrovirus. J. Gen. Virol..

[42] Wolff J.L., Valicente F.H., Martins R., Oliveira J.V., Zanotto P.M. (2008). Analysis of the genome of Spodoptera frugiperda nucleopolyhedrovirus (SfMNPV-19) and of the high genomic heterogeneity in group II nucleopolyhedroviruses. J. Gen. Virol..

[43] del Rincón-Castro M.C., Ibarra J.E. (1997). Genotypic divergence of three single nuclear polyhedrosis virus (SNPV) strains from the cabbage looper, *Trichoplusia ni*. Biochem. Syst. Ecol..

[44] van Oers M.M., Herniou E.A., Usmany M., Messelink G.J., Vlak J.M. (2004). Identification and characterization of a DNA photolyase-containing baculovirus from *Chrysodeixis chalcites*. Virology.

[45] Fielding B.C., Davison S. (1999). The characterization and phylogenetic relationship of the Trichoplusia ni single capsid nuclear polyhedrosis virus polyhedrin gene. Virus Genes.

[46] Kumar C.M.S., Jacob T.K., Devasahayam S., D’Silva S., Jinsha J., Rajna S. (2015). Occurrence and characterization of a tetrahedral nucleopolyhedrovirus from *Spilarctia obliqua* (Walker). J. Invert. Path..

[47] Cheng X.W., Carner G.R., Fescemyer H.W. (1998). Polyhedrin sequence determines the tetrahedral shape of occlusion bodies in Thysanoplusia orichalcea single-nucleocapsid nucleopolyhedrovirus. J. Gen. Virol..

[48] Todd J.W., Passarelli A.L., Miller L.K. (1995). Eighteen baculovirus genes, including lef-11, p35, 39K, and p47, support late gene expression. J. Virol..

[49] Rapp J.C., Wilson J.A., Miller L.K. (1998). Nineteen baculovirus open reading frames, including LEF-12, support late gene expression. J. Virol..

[50] Liu X., Yin F., Zhu Z., Hou D., Wang J., Zhang L., Wang M., Wang H., Hu Z., Deng F. (2014). Genomic sequencing and analysis of Sucra jujuba nucleopolyhedrovirus. PLoS ONE.

[51] Becker D., Knebel-Morsdorf D. (1993). Sequence and temporal appearance of the early transcribed baculovirus gene HE65. J. Virol..

[52] Harrison R.L., Mowery J.D., Rowley D.L., Bauchan G.R., Theilmann D.A., Rohrmann G.F., Erlandson M.A. (2018). The complete genome sequence of a third distinct baculovirus isolated from the true armyworm, *Mythimna unipuncta*, contains two copies of the lef-7 gene. Virus Genes.

[53] Li L., Donly C., Li Q., Willis L.G., Keddie B.A., Erlandson M.A., Theilmann D.A. (2002). Identification and genomic analysis of a second species of nucleopolyhedrovirus isolated from *Mamestra configurata*. Virology.

[54] Bourner T.C., Cory J.S. (2004). Host range of an NPV and a GV isolated from the common cutworm, *Agrotis segetum*: Pathogenicity within the cutworm complex. Biol. Control.

[55] Wennmann J.T., Gueli Alletti G., Jehle J.A. (2015). The genome sequence of Agrotis segetum nucleopolyhedrovirus B (AgseNPV-B) reveals a new baculovirus species within the *Agrotis* baculovirus complex. Virus Genes.

[56] Harrison R.L., Mowery J.D., Bauchan G.R., Theilmann D.A., Erlandson M.A. (2019). The complete genome sequence of a second alphabaculovirus from the true armyworm, *Mythimna unipuncta*: Implications for baculovirus phylogeny and host specificity. Virus Genes.

[57] Herniou E.A., Jehle J.A. (2007). Baculovirus phylogeny and evolution. Curr. Drug Targets.

[58] Guarino L.A., Mistretta T.A., Dong W. (2002). Baculovirus *lef-12* is not required for viral replication. J. Virol..

[59] Lauzon H.A., Jamieson P.B., Krell P.J., Arif B.M. (2005). Gene organization and sequencing of the Choristoneura fumiferana defective nucleopolyhedrovirus genome. J. Gen. Virol..

[60] Oliveira J.V., Wolff J.L., Garcia-Maruniak A., Ribeiro B.M., de Castro M.E., de Souza M.L., Moscardi F., Maruniak J.E., Zanotto P.M. (2006). Genome of the most widely used viral biopesticide: Anticarsia gemmatalis multiple nucleopolyhedrovirus. J. Gen. Virol..

[61] Brito A.F., Braconi C.T., Weidmann M., Dilcher M., Alves J.M., Gruber A., Zanotto P.M. (2015). The pangenome of the Anticarsia gemmatalis multiple nucleopolyhedrovirus (AgMNPV). Genome Biol. Evol..

[62] Harrison R.L. (2013). Concentration- and time-response characteristics of plaque isolates of Agrotis ipsilon multiple nucleopolyhedrovirus derived from a field isolate. J. Invertebr. Pathol..

