# Supplementary material for: A Novel Alphabaculovirus from the Soybean Looper, Chrysodeixis includens, that Produces Tetrahedral Occlusion Bodies and Encodes Two Copies of he65
Source: Viruses. 2019 Jun 26;11(7):579. doi: 10.3390/v11070579 (PMC6669638; doi:10.3390/v11070579)
Supplement: Supplementary file 1 [file viruses-11-00579-s001.zip › Table S2 ChinNPV#1 ORFs.docx]

**Supplementary Table 2. ChinNPV #1 ORFs**

| ORF | Name | Position | aa | PsinNPV-IE | ChchNPV | TnSNPV | AgipMNPV | Notes/AcMNPV homologues |
| --- | --- | --- | --- | --- | --- | --- | --- | --- |
| 1 | *polh* | 1🡪741 | 246 | 1 (98.4%) | 1 (98.8%) | 1 (100%) | 1 (89.8%) | *ac8*; also shares 99.6% sequence identity with ThorSNPV polyhedrin |
| 2 | *orf1629; pp78/83* | 1901🡨738 | 387 | 2 (38.9%) | 2 (39.1%) | 2 (33.8%) | 2 (36.9%) | *ac9* |
| 3 | *pk-1* | 1925🡪2746 | 273 | 3 (58.2%) | 3 (56.8%) | 3 (56.8%) | 3 (55.4%) | *ac10* |
| 4 | *hoar* | 4995🡨2812 | 727 | 4 (NSS) | 4 (25.9%) | 4 (NSS) | 4 (NSS) |  |
| 5 |  | 5673🡨5404 | 89 | - | - | - | - | Match with Hyposidra talaca NPV ORF5 (e = 0.022) |
| 6 |  | 5848🡪7107 | 419 | - | - | - | - |  |
| 7 | *pif-5/odv-e56* | 7365🡪8435 | 356 | 9 (73.1%) | 7 (73.9%) | 8 (73.1%) | 9 (57.9%) | *ac148* |
| 8 | *me53* | 9807🡨8698 | 369 | 10 (55.0%) | 8 (55.8%) | 9 (57.9%) | 10 (44.6%) | *ac139* |
| 9 | *exon0* | 10111🡪11016 | 301 | 11 (46.4%) | 10 (49.8%) | 10 (47.5%) | 161 (47.7%) | *ac141* |
| 10 | *p49* | 11022🡪12443 | 473 | 12 (75.9%) | 11 (75.5%) | 11 (76.1%) | 160 (58.5%) | *ac142* |
| 11 | *odv-e18* | 12464🡪12706 | 80 | 13 (81.9%) | 12 (80.7%) | 12 (84.3%) | 158 (58.3%) | *ac143* |
| 12 | *odv-ec27* | 12741🡪13589 | 282 | 14 (54.6%) | 13 (54.2%) | 13 (55.4%) | 156 (52.3%) | *ac144* |
| 13 | *chtB1* | 13603🡪13884 | 93 | 15 (67.7%) | 14 (67.7%) | 14 (66.7%) | 155 (54.8%) | *ac145* |
| 14 | *ep23* | 14576🡨13950 | 208 | 16 (57.1%) | 15 (56.2%) | 15 (55.7%) | 154 (45.1%) | *ac146* |
| 15 | *ie-1* | 14616🡪17021 | 801 | 17 (38.6%) | 16 (41.6%) | 16 (40.8%) | 153 (37.0%) | *ac147* |
| 16 | *pif-0*/*p74* | 17320🡪19293 | 657 | 18 (71.9%) | 17 (71.3%) | 17 (70.2%) | 151 (60.9%) | *ac138* |
| 17 | *p10* | 19592🡨19338 | 84 | 19 (71.1%) | 18 (69.0%) | 18 (73.7%) | 150 (62.3%) | *ac137* |
| 18 | *p26a* | 20590🡨19655 | 311 | 20 (61.1%) | 19 (55.0%) | 19 (59.9%) | 149 (64.2%) | *ac136* |
| 19 |  | 20726🡪20971 | 81 | 21 (56.6%) | 20 (58.5%) | 20 (56.6%) | 148 (56.6%) |  |
| 20 | *lef-6* | 21838🡨21038 | 266 | 22 (61.0%) | 21 (64.0%) | 21 (65.3%) | 147 (56.9%) | *ac28* |
| 21 | *dbp* | 22809🡨21814 | 331 | 23 (31.1%) | 22 (28.6%) | 22 (31.6%) | 146 (31.0%) | *ac25* |
| 22 |  | 24130🡨23546 | 194 | 26 (55.5%) | 25 (62.3%) | 24 (63.3%) | 143 (54.8%) |  |
| 23 | *v-ubi* | 24250🡪24498 | 82 | 27 (85.5%) | 26 (84.2%) | 25 (85.5%) | 142 (80.3%) | *ac35* |
| 24 |  | 24485🡪24694 | 69 | 28 (35.1%) | 27 (37.5%) | 25b (36.1%) | 141 (35.5%) |  |
| 25 | *pp31/39k* | 25741🡨24755 | 328 | 29 (36.3%) | 28 (38.2%) | 26 (36.5%) | 139 (40.0%) | *ac36* |
| 26 | *lef-11* | 26123🡨25716 | 115 | 30 (57.9%) | 29 (60.0%) | 27 (59.2%) | 138 (57.0%) | *ac37* |
| 27 | *bv-e31; adprase* | 26752🡨26009 | 247 | 31 (63.7%) | 30 (57.0%) | 28 (55.8%) | 137 (63.9%) | *ac38* |
| 28 |  | 27473🡨26913 | 186 | - | 32 (20.9%) | 30 (22.8%) | 136 (20.4%) |  |
| 29 | *p47* | 28789🡨27569 | 406 | 34 (67.1%) | 33 (68.0%) | 31 (66.3%) | 134 (62.8%) | *ac40* |
| 30 | *lef-12* | 28900🡨29727 | 375 | - | - | - | - | *ac41*; Top match is SujuNPV lef-12 (36.1%) |
| 31 |  | 29666🡪29854 | 62 | - | 35 (42.0%) | 32 (38.0%) | 132 (41.8%) |  |
| 32 | *bro* | 31276🡨29951 | 441 | - | - | - | - | Best match: Bro-N domain-containing protein [Bodo saltans virus], 34.3% |
| 33 | *lef-8* | 33980🡨31359 | 873 | 37 (63.4%) | 37 (63.4%) | 33 (64.0%) | 130 (65.1%) | *ac50* |
| 34 | *bjdp* | 34004🡪35008 | 334 | 38 (46.6%) | 38 (47.4%) | 34 (46.6%) | 129 (36.1%) | *ac51* |
| 35 | *iap-3* | 35817🡨35053 | 154 | 39 (29.9%) | 39 (28.2%) | 35 (30.4%) | 128 (26.2%) |  |
| 36 |  | 36618🡨36079 | 179 | 41 (31.7%) | 40 (30.7%) | 37 (32.1%) | 127 (29.0%) |  |
| 37 |  | 36680🡪37096 | 138 | 42 (61.3%) | 41 (60.6%) | 38 (60.6%) | 126 (49.3%) | *ac53* |
| 38 |  | 38274🡨37150 | 374 | 43 (37.8%) | 42 (40.2%) | 39 (40.2%) | 125 (39.7%) |  |
| 39 |  | 38523🡨38296 | 75 | 44 (55.8%) | 43 (51.9%) | 40 (55.8%) | 124 (45.5%) |  |
| 40 | *lef-10* | 38483🡪38707 | 74 | 45 (58.7%) | 44 (53.9%) | 41 (58.7%) | 123 (61.2%) | *ac53a* |
| 41 | *vp1054* | 38571🡪39578 | 335 | 46 (58.8%) | 45 (62.3%) | 42 (62.6%) | 122 (60.8%) | *ac54* |
| 42 |  | 39694🡪39912 | 72 | 47 (55.1%) | 46 (49.4%) | 43 (45.1%) | 121 (52.8%) | *ac55* |
| 43 |  | 39854🡪40165 | 103 | 48 (44.0%) | 47 (50.5%) | 44 (47.2%) | 120 (35.7%) | *ac56* |
| 44 |  | 40395🡪40958 | 187 | 49 (53.3%) | 48 (54.9%) | 45 (55.2%) | 119 (43.3%) | *ac57* |
| 45 | *chaB1* | 41473🡨40967 | 168 | 50 (57.3%) | 49 (60.2%) | 46 (58.6%) | 118 (63.4%) | *ac58/59* |
| 46 | *chaB2* | 41799🡨41518 | 93 | 51 (59.4%) | 50 (56.5%) | 47 (59.4%) | 117 (53.7%) | *ac60* |
| 47 | *fp25k* | 42897🡨42154 | 247 | 52 (80.6%) | 51 (80.6%) | 48 (79.4%) | 114 (74.1%) | *ac61* |
| 48 | *lef-9* | 42967🡪44460 | 497 | 53 (76.3%) | 52 (77.3%) | 49 (77.3%) | 113 (69.5%) | *ac62* |
| 49 |  | 44987🡪45244 | 85 | 55 (88.2%) | 56 (89.4%) | 52 (88.2%) | 108 (77.6%) | *ac76* |
| 50 |  | 45259🡪45645 | 128 | 56 (67.2%) | 57 (67.7%) | 53 (66.9%) | 107 (56.3%) | *ac75* |
| 51 | *dnapol* | 49050🡨45721 | 1109 | 57 (55.4%) | 58 (58.9%) | 54 (59.2%) | 106 (52.8%) | *ac65* |
| 52 | *desmoplakin* | 49052🡨51184 | 710 | 58 (46.3%) | 59 (46.7%) | 55 (48.6%) | 105 (43.3%) | *ac66* |
| 53 | *lef-3* | 53103🡨51442 | 553 | 59 (32.6%) | 60 (34.4%) | 56 (32.1%) | 104 (31.3%) | *ac67* |
| 54 | *pif-6* | 53102🡪53515 | 137 | 60 (75.9%) | 61 (77.0%) | 57 (73.0%) | 103 (69.2%) | *ac68* |
| 55 | *iap-2* | 53534🡪54397 | 287 | 61 (52.2%) | 62 (50.7%) | 58 (49.4%) | 101 (44.1%) | *ac71* |
| 56 | *p26b* | 54457🡪55173 | 238 | 62 (60.8%) | 63 (64.3%) | 59 (63.2%) | 100 (42.7%) | *ac136* |
| 57 |  | 55911🡨55222 | 229 | - | - | - | 111 (29.1%) |  |
| 58 | *v-cath* | 57032🡨56010 | 340 | 63 (77.3%) | 64 (72.0%) | 60 (73.0%) | 23 (58.2%) | *ac127* |
| 59 | *chitinase* | 57121🡪58815 | 564 | 64 (72.5%) | 65 (73.9%) | 61 (72.4%) | 27 (66.1%) | *ac126* |
| 60 | *gp37* | 58882🡪59709 | 375 | 67 (76.4%) | 67 (79.3%) | 64 (76.4%) | 30 (67.2%) | *ac64* |
| 61 | *phr-2* | 60021🡪61565 | 514 | 68 (44.6%) | 72 (57.9%) | 65 (47.7%) | - |  |
| 62 |  | 61970🡨61749 | 73 | 70 (37.0%) | 71 (30.0%) | 66 (36.0%) | - | *ac111* |
| 63 | *he65a* | 62078🡪62779 | 233 | 71 (56.4%) | 73 (49.6%) | - | 25 (45.3%) | *ac105* |
| 64 | *vlf-1* | 64199🡨63012 | 395 | 74 (83.5%) | 76 (89.2%) | 70 (89.5%) | 99 (73.4%) | *ac77* |
| 65 |  | 64555🡨64211 | 114 | 75 (40.7%) | 77 (41.6%) | 71 (38.6%) | 98 (42.0%) | *ac78* |
| 66 | *gp41* | 65536🡨64586 | 316 | 76 (77.5%) | 78 (77.7%) | 72 (75.4%) | 97 (58.2%) | *ac80* |
| 67 |  | 66200🡨65539 | 224 | 77 (70.0%) | 79 (77.2%) | 74 (76.2%) | 96 (62.5%) | *ac81* |
| 68 | *tlp-20* | 66777🡨66109 | 222 | 78 (46.6%) | 80 (49.2%) | 75 (55.4%) | 95 (44.3%) | *ac82* |
| 69 | *vp91/p95* | 66746🡨69112 | 788 | 79 (46.0%) | 81 (45.1%) | 76 (45.6%) | 94 (44.4%) | *ac83* |
| 70 | *he65b* | 69222🡪70028 | 268 | 71  (73.6%) | 73 (70.8%) | - | 25 (39.3%) | *ac105* (48.2% amino acid sequence identity with he65a) |
| 71 |  | 70641🡨70063 | 192 | 73 (40.6%) | 75 (58.7%) | 69 (54.3%) | - | *ac84* |
| 72 | *vp39* | 71776🡨70736 | 346 | 80 (42.5%) | 82 (44.3%) | 77 (44.3%) | 92 (45.6%) | *ac89* |
| 73 | *lef-4* | 71775🡪73166 | 462 | 81 (54.2%) | 83 (61.6%) | 78 (61.6%) | 91 (54.8%) | *ac90* |
| 74 | *p33* | 73999🡨73232 | 255 | 82 (65.7%) | 84 (64.1%) | 79 (66.1%) | 89 (59.4%) | *ac92* |
| 75 | *p18* | 74010🡨74480 | 156 | 83 (80.0%) | 85 (80.0%) | 80 (80.0%) | 88 (72.9%) | *ac93* |
| 76 | *odv-e25* | 74477🡨75133 | 218 | 84 (74.8%) | 86 (75.3%) | 81 (77.1%) | 87 (72.4%) | *ac94* |
| 77 | *helicase* | 79073🡨75252 | 1273 | 85 (48.0%) | 87 (48.2%) | 82 (54.7%) | 86 (51.4%) | *ac95* |
| 78 | *pif-4; odv-e28* | 79039🡪79557 | 172 | 86 (65.6%) | 88 (67.5%) | 83 (66.2%) | 85 (71.7%) | *ac96* |
| 79 | *38k* | 80738🡨79809 | 309 | 89 (57.7%) | 91 (59.4%) | 86 (60.0%) | 81 (56.1%) | *ac98* |
| 80 | *lef-5* | 80631🡪81470 | 279 | 90 (57.1%) | 92 (62.6%) | 87 (64.4%) | 80 (55.6%) | *ac99* |
| 81 | *p6.9* | 81727🡨81467 | 86 | 91 (NSS) | 93 (NSS) | 88 (NSS) | 79 (NSS) | *ac100* |
| 82 | *bv/odv-c42; p40* | 82921🡨81788 | 377 | 92 (58.5%) | 94 (57.9%) | 89 (58.1%) | 78 (53.9%) | *ac101* |
| 83 | *p12* | 83320🡨82949 | 123 | 93 (60.2%) | 95 (51.2%) | 90 (57.1%) | 77 (49.4%) | *ac102* |
| 84 | *p48/p45* | 84446🡨83304 | 380 | 94 (73.3%) | 96 (74.8%) | 91 (75.3%) | 76 (73.7%) | *ac103* |
| 85 | *vp80* | 84490🡪86277 | 595 | 95 (44.9%) | 97 (45.7%) | 92 (50.9%) | 75 (29.1%) | *ac104* |
| 86 | *pif-7* | 86279🡪86446 | 55 | Not annotated (77.3%) | 98 (67.6%) | 93 (76.7%) | 74 (58.0%) | *ac110* |
| 87 | *odv-ec43* | 86448🡪87521 | 357 | 96 (77.2%) | 99 (76.8%) | 94 (76.8%) | 73 (63.3%) | *ac109* |
| 88 |  | 87543🡪87818 | 92 | 97 (65.9%) | 100 (64.8%) | 95 (63.6%) | 72 (61.7%) | *ac108* |
| 89 | *odv-e66* | 89859🡨87865 | 664 | 98 (64.6%) | 101 (67.3%) | 96 (67.1%) | 71 (56.8%) | *ac46* |
| 90 | *p13* | 90767🡨89910 | 285 | 99 (54.8%) | 102 (54.2%) | 97 (54.5%) | 70 (60.4%) |  |
| 91 |  | 92092🡨91391 | 233 | 104 (75.5%) | 107 (73.1%) | 102 (73.8%) | 62 (63.2%) | *ac106/107* |
| 92 | *parg* | 93699🡨92128 | 523 | 105 (43.1%) | 108 (45.6%) | 103 (45.1%) | 61 (29.4%) |  |
| 93 | *pif-3* | 94323🡨93709 | 204 | 107 (40.5%) | 110 (41.2%) | 105 (42.0%) | 59 (48.0%) | *ac115* |
| 94 |  | 95486🡨94557 | 309 | 106 (30.3%) | 109 (33.9%) | 104 (33.9%) | - |  |
| 95 |  | 95633🡪96214 | 193 | 109 (61.1%) | 112 (63.9%) | 107 (62.5%) | - |  |
| 96 |  | 98082🡨96343 | 579 | - | 113 (28.9%) | - | - |  |
| 97 | *sod* | 98335🡪98790 | 151 | 110 (77.5%) | 115 (75.3%) | 109 (75.3%) | 57 (73.5%) | *ac31* |
| 98 | *dut* | 99572🡨98841 | 243 | 114 (32.3%) | 119 (35.4%) | - | 64 (29.2%) |  |
| 99 | *pep* | 99778🡪100716 | 312 | 115 (74.8%) | 121 (75.2%) | 113 (80.5%) | 54 (72.3%) | *ac131* |
| 100 |  | 100876🡪101307 | 143 | 117 (36.3%) | 123 (37.9%) | 115 (37.9%) | 51 (33.3%) |  |
| 101 |  | 101304🡪102434 | 376 | 118 (58.5%) | 124 (59.3%) | 116 (58.5%) | - |  |
| 102 |  | 103722🡨102496 | 408 | 119 (43.1%) | 125 (43.2%) | 117 (41.8%) | 50 (37.7%) |  |
| 103 |  | 103724🡪104068 | 104 | 120 (46.3%) | 126 (50.5%) | 118 (50.5%) | 49 (36.3%) |  |
| 104 | *alk-exo; an* | 104140🡪105375 | 411 | 121 (44.6%) | 127 (43.9%) | 119 (44.5%) | 47 (39.6%) | *ac133* |
| 105 |  | 106185🡨105415 | 256 | 122 (52.6%) | 128 (55.8%) | 120 (53.4%) | 46 (38.0%) |  |
| 106 | *fgf* | 106438🡪107508 | 356 | 123 (37.4%) | 130 (43.2%) | 122 (38.7%) | 45 (31.7%) | *ac32* |
| 107 | *pif-1* | 109120🡨107555 | 521 | 124 (54.4%) | 131 (53.8%) | 123 (53.3%) | 43 (52.0%) | *ac119* |
| 108 |  | 109847🡨109197 | 216 | - | - | - | - |  |
| 109 | *gp16* | 110205🡨109909 | 98 | 126 (58.1%) | 133 (60.2%) | 125 (59.1%) | 14 (45.2%) | *ac130* |
| 110 | *p24 capsid* | 110962🡨110225 | 245 | 127 (55.0%) | 134 (57.0%) | 126 (57.1%) | 15 (51.0%) | *ac129* |
| 111 |  | 111057🡪111473 | 138 | 128 (38.6%) | 135 (37.5%) | 127 (39.4%) | - |  |
| 112 | *lef-2* | 111409🡪112065 | 218 | 129 (54.6%) | 136 (53.0%) | 128 (55.6%) | 17 (43.6%) | *ac6* |
| 113 | *38.7k* | 113298🡨112219 | 359 | 130 (40.8%) | 137 (44.1%) | 129 (43.4%) | 21 (40.0%) | *ac13* |
| 114 | *lef-1* | 113994🡨113341 | 217 | 131 (56.0%) | 138 (54.5%) | 130 (54.2%) | 22 (48.1%) | *ac14* |
| 115 |  | 113993🡪114520 | 175 | 132 (75.9%) | 139 (58.1%) | 131 (74.7%) | - |  |
| 116 | *dna ligase 3* | 116435🡨114591 | 614 | - | - | - | - | No matches with viral sequences. Top match is with DNA ligase 3 [Trichoplusia ni] |
| 117 | *chtB2* | 116817🡨116524 | 97 | - | - | - | - | *ac150* |
| 118 | *egt* | 117101🡪118675 | 524 | 134 (77.4%) | 141 (76.9%) | 133 (79.0%) | 32 (68.3%) | *ac15* |
| 119 |  | 118870🡪119424 | 184 | 135 (48.9%) | 142 (55.7%) | 135 (57.4%) | 33 (31.1%) |  |
| 120 |  | 122355🡨119662 | 897 | 136 (45.6%) | 143 (47.4%) | 136 (46.7%) | 35 (35.6%) |  |
| 121 | *pkip-1* | 122672🡪123202 | 176 | 137 (45.2%) | 146 (44.0%) | 139 (45.2%) | 38 (38.0%) | *ac24* |
| 122 |  | 123588🡨123238 | 116 | - | - | - | 40 (32.5%) |  |
| 123 | *arif-1* | 124549🡨123659 | 296 | 138 (33.6%) | 147 (36.4%) | 140 (36.5%) | 41 (39.1%) | *ac20/21* |
| 124 | *pif-2* | 124544🡪125710 | 388 | 139 (67.5%) | 148 (66.5%) | 141 (65.9%) | 42 (67.8%) | *ac22* |
| 125 | *efp* | 127780🡨125759 | 673 | 140 (62.1%) | 150 (61.5%) | 143 (61.7%) | 12 (46.4%) | *ac23* |
| 126 | *rr1* | 130332🡨128011 | 773 | 141 (40.1%) | 151 (41.5%) | 144 (41.3%) | 163 (45.3%) |  |
